# Supplementary material for: Smart thrombosis inhibitors without bleeding side effects via charge tunable ligand design
Source: Nat Commun. 2023 Apr 26;14:2177. doi: 10.1038/s41467-023-37709-0 (PMC10133246; doi:10.1038/s41467-023-37709-0)
Supplement: Supplementary file 1 — Supplementary Information [file 41467_2023_37709_MOESM1_ESM.pdf]

Supplementary Information

Smart Thrombosis Inhibitors Without Bleeding Side Effects via Charge Tunable Ligand Design

Chanel C. La,[1,2] Stephanie A Smith,[3] Sreeparna Vappala,[1,4] Reheman Adili,[5,13] Catherine E. Luke,[6] Srinivas Abbina,[1,4] Haiming D. Luo,[1,2] Irina Chafeeva,[1,4] Matthew Drayton,[1,4] Louise A. Creagh,[7,8] Maria de Guadalupe Jaraquemada-Peláez,[2] Nicole Rhoads,[9] Manu Thomas Kalathottukaren,[1,4] Peter K. Henke[6], Suzana K. Straus,[2] Caigan Du[10], Edward M. Conway[1,4,11,12], Michael Holinstat,[5] Charles A. Haynes,[7,8] James H. Morrissey,[3,*] Jayachandran N. Kizhakkedathu[1,2,4,12*]

1. Centre for Blood Research, Life Sciences Institute, University of British Columbia, Canada
2. Department of Chemistry, University of British Columbia, Canada
3. Department of Biological Chemistry, University of Michigan Medical School, United States.
4. Department of Pathology and Laboratory Medicine, University of British Columbia, Canada.
5. Department of Pharmacology, University of Michigan Medical School, United States.
6. Department of Surgery, Section of Vascular Surgery, University of Michigan Medical School, United States
7. Department of Chemical and Biological Engineering, University of British Columbia, Canada
8. Michael Smith Laboratories, University of British Columbia, Canada
9. Bloodworks Research Institute, 1551 Eastlake Avenue E.; Ste.100, Seattle, WA, 98102, United States
10. Department of Urological Sciences, University of British Columbia, Canada
11. Department of Medicine, University of British Columbia, Canada
12. The School of Biomedical Engineering, University of British Columbia, Canada
13. Present address: Bloodworks Research Institute, 1551 Eastlake Avenue E., Ste. 100, Seattle, WA 98102, United States

*To whom the correspondence should be addressed

Jayachandran N. Kizhakkedathu (email: jay@pathology.ubc.ca)

or

James H. Morrissey (email: jhmorris@umich.edu)

Supplementary Information Inventory

Items	Page No.
Title page	i
Supplementary Methods	iii-viii
Supplementary Figure 1	9
Supplementary Figure 2	10
Supplementary Figure 3	11
Supplementary Figure 4	12
Supplementary Figure 5	13
Supplementary Figure 6	14
Supplementary Figure 7	14
Supplementary Figure 8	15
Supplementary Figure 9	16
Supplementary Figure 10	17
Supplementary Figure 11	18
Supplementary Figure 12	19
Supplementary Figure 13	19
Supplementary Figure 14	20
Supplementary Figure 15	21
Supplementary Figure 16	21
Supplementary Figure 17	22
Supplementary Figure 18	22
Supplementary Figure 19	23
Supplementary Table 1	24
Supplementary Table 2	25
Supplementary Table 3	25
Supplementary Table 4	26

Supplementary Methods

Experimental Details

Materials and Reagents

Human fibrinogen, polyethyleneimine (PEI, 25 kDa), thrombin, protamine sulfate and N-2-hydroxyethyl piperazine-N'-2-ethanesulfonic acid (HEPES) were from Sigma Aldrich. Recombinant tissue factor (TF, Innovin) was from Dade-Behring. Polystyrene 96-well microplates (Costar) used for clotting assays were from Corning. A Spectramax microplate reader (Molecular Devices, CA, USA) was used. Reagents used for calibrated automated thrombography, including the thrombin calibrator, Flu-Ca solution and Immulon microplates were purchased from Diagnostica Stago. Buffer for biological assays was prepared with 20 mM HEPES with 150 mM NaCl, pH 7.4 unless otherwise stated. The protocols for synthesis of UHRA 8, UHRA 10[31-33] and MPIs studied are provided and physical characteristics are shown in Table 1. BD Vacutainer® Citrate Tubes containing 3.2 % buffered sodium citrate solution were purchased from Becton, Dickinson and Company (New Jersey, USA).

All other chemicals were purchased and used without further purification, unless indicated otherwise. Glycidol was purified by vacuum distillation and stored at 4 °C using molecular sieves (5 Å). Cellulose ester dialysis membranes were obtained from Spectra/Por Biotech (Rancho Dominguez, CA, USA). $CDCl_3$ or D_2O (Cambridge Isotope Laboratories, Andover MA) were used as solvents, with the relevant solvent peak as reference. Chelating resin (Chelex® 100) was purchased from Bio-Rad.

Techniques

A NE-1000 Programmable Single Syringe Pump (Farmingdale, NY) was used for chemical synthesis and polymerization. Absolute molecular weights of the polymers were determined by GPC on a Waters 2695 separation module fitted with a DAWN EOS multi-angle laser light scattering (MALLS) detector coupled with Optilab DSP refractive index detector, both from Wyatt Technology. GPC analysis was performed using Waters ultrahydrogel 7.8 x 300 columns (guard, 250 and 120) and 0.1 N $NaNO_3$ at pH 7.4 using 10 mM phosphate buffer as the mobile phase. ^1H NMR spectra were recorded on a Bruker Advance 300 MHz NMR spectrometer and Bruker Advance 400 MHz NMR spectrometer.

MPI synthesis, Modification and Conjugation

Synthesis of $mPEG_{350}$ epoxide

$mPEG_{350}$ (110 g, 314 mmol, 1.0 eq.) was added to a 500 mL round bottom flask with a magnetic stir bar. To this, crushed NaOH pellets (39 g, 940 mmol, 3.0 eq.) were added and the mixture was left to stir for 24 hours. The reaction mixture was then cooled to 0°C and epichlorohydrin (53 mL, 658 mmol, 2.0 eq.) was slowly added over the course of 3 hours. The reaction was kept cool by replenishing the ice bath over the course of the slow addition. After complete addition of epichlorohydrin, the reaction mixture was stirred for an additional 24 hours and slowly warmed to room temperature. Once complete, the reaction was quenched with MeOH and the remaining salts filtered off with DCM. Crude product was concentrated under vacuum and dried on a vacuum drying line for 2 days to evaporate unreacted epichlorohydrin, affording the pure $mPEG_{350}$ epoxide in 90% yield.

^{1}H NMR (300 MHz, chloroform-*d*): δ 3.78 (dd, *J* = 11.7, 3.1 Hz, 1H), 3.73 – 3.51 (m, 33H), 3.47 – 3.35 (m, 4H), 3.16 (dq, *J* = 6.1, 3.1 Hz, 1H), 2.79 (t, *J* = 4.6 Hz, 1H), 2.61 (dd, *J* = 5.0, 2.7 Hz, 1H).

General procedure for HPG-PEG synthesis

A three-neck round bottom flask was cooled under vacuum and filled with argon. To this, 1,1,1-tris(hydroxymethyl)propane **(1)** (TMP, 167 mg) and potassium methylate (25 wt% solution in methanol, 0.110 mL) were added and stirred for 30 minutes. Methanol was removed under high vacuum for 4 hours. The flask was heated to 95°C and distilled glycidol (3.8 mL) was added over a period of 15 hours. After complete addition of glycidol, the reaction mixture was stirred for an additional 12 hours. Then, $mPEG_{350}$-epoxide (10.5 mL) was added over a period of 12 hours at 95°C. The reaction mixture was stirred for an additional 4 hours. The reaction was cooled to room temperature, quenched with methanol, then passed through Amberlite IR-120H resin to remove the potassium ions and twice precipitated from diethyl ether. The polymer was then dissolved in water and dialyzed using a membrane with MW cutoff of 3500 Da for 3 days with periodic changes of the water in which the dialysis bag was kept.

^{1}H NMR (300 MHz, chloroform-*d*) δ 4.04 – 3.40 (m, 50H), 3.38 (s, 3H).

mPEG content (by ^{1}H NMR): 25 mol%; Polyglycerol: 75 mol%.

GPC-MALLS (0.1 M $NaNO_3$): M_n 23 500; M_w/M_n 1.3. or Mn 21 500; M_w/M_n 1.25

For polymers with a molecular weight of 10 kDa, the same synthetic procedure was followed, except that a higher initiator:monomer ratio was used. In this case, 1,1,1-tris(hydroxymethyl)propane **(1)** (TMP, 268 mg) and potassium methylate (25 wt% solution in methanol, 0.178 mL) were added to a three-neck round bottom flask that was cooled under vacuum and filled with argon prior to the addition of the mixture. The solution was stirred for 30 minutes. Methanol was removed under high vacuum for 4 hours, as described above. The flask was heated to 95°C and distilled glycidol (5.71 mL) was added over a period of 23 hours. After complete addition of glycidol, the reaction mixture was stirred for an additional 3 hours. Then, $mPEG_{350}$-epoxide **(4)** (12.8 mL) was added over a period of 12 hours at 95°C. The subsequent steps are identical to those described above. Dialysis in water was performed with a membrane MW cutoff of 1000 Da.

^{1}H NMR (300 MHz, chloroform-*d*) δ 4.04 – 3.40 (m, 50H), 3.38 (s, 3H).

mPEG content (by ^{1}H NMR): 27.5 mol%; Polyglycerol: 72.5 mol%.

GPC-MALLS (0.1 M $NaNO_3$): M_n 10 360; M_w/M_n 1.2.

General procedure for HPG-mPEG-OTs synthesis

This reaction was carried out under an inert gas atmosphere and exclusion of water. HPG-mPEG (200 mg) was dissolved in pyridine (5 mL) in a three necked 100 mL flask, equipped with a thermometer and magnetic stirrer. The solution was cooled to 0°C by means of an ice/NaCl bath; then, a solution of TsCl (1.2 eq. of target average number of groups) in pyridine (5 mL) was added dropwise so that the temperature did not exceed 5°C. The brown mixture was stirred for 16 hours as the reaction was allowed to warm to room temperature. The solvent was then removed *in vacuo*. The resulting mixture was then dialyzed for 3 days against water to give a honey-like product.

^{1}H NMR (400 MHz, chloroform-*d*): δ 7.78 (m, 0.7H), 7.34 (m, 0.7H), 3.98 – 3.39 (m, 48H), 3.35 (s, 3H), 2.45 (s, 1.05H).

General procedure for HPG-mPEG-NH_2 synthesis

In a 50 mL one-necked flask, equipped with a reflux condenser and magnetic stirrer, HPG-mPEG-OTs (200 mg, 0.20 mmol OTs-groups) was dissolved in anhydrous 1,4-dioxane (10 mL). After addition of amine CBG (1 mmol, 5.0 eq.) (diethylene triamine (CAS 111-40-0) (MPI 1, MPI 2, MPI 3, MPI 6, MPI 7) or bis (2-aminopropyl) amine (CAS 56-18-8) (MPI 4, MPI 5, MPI 8, MPI 9) the resulting suspension was heated to reflux at 115 °C for 24 hours. After cooling the reaction mixture, the mixture was dialyzed against water for three days with periodic changes of the water to give a brown honey-like product.

^{1}H NMR (300 MHz, chloroform-*d*): δ 3.89 – 3.43 (m, 35H), 3.38 (s, 3H), 2.87 (m, 9H).

General procedure for HPG-mPEG-NMe_2 (MPI) synthesis

Amine functionalized HPG-mPEG (200 mg, 24 CBG per polymer) was transferred to a 20 mL side-necked flask. The reaction mixture was dissolved in deionized water at room temperature. 10 mL of a formic acid/formaldehyde (1:1) mixture was added dropwise to the reaction flask. The reaction was stirred and heated to reflux at 110°C for 24 hours. After cooling, the reaction mixture was dialyzed against water for two days with periodic changes in water until the pH of the water was neutral. Upon lyophilizing to remove excess water, MPI was collected.

^{1}H NMR (300 MHz, chloroform-*d*): δ 8.11 (s), 4.11 – 3.19 (m), 3.19 – 2.95 (m), 2.78 – 1.94 (m).

Physical Characterizations

Conductometric measurements

The time allowed for equilibration was 15 seconds for conductometry titrations. A solution of MPIs in water (0.1 mM) was acidified dropwise with 1.0 M HCl and titrated with carbonate-free NaOH (0.5012 M) that was standardized against freshly recrystallized potassium hydrogen phthalate. The temperature was kept constant at 25ºC with a warm water bath. Titration curves were manually fitted to calculate the proton concentration.

Potentiometric measurements

All data were collected in triplicate. Prior to each titration, the electrode was calibrated using a standard HCl solution. Calibration data were calculated to obtain parameters E_0 and pK_w. Time allowed for equilibration was 10 minutes for pK_a titrations. Solutions were titrated with carbonate-free NaOH (0.141 M) that was standardized against freshly recrystallized potassium hydrogen phthalate. Protonation equilibria of the CBG were studied by NaOH titrations of a solution containing (CBG or MPIs) 1.1 x 10^{-3} M at 25 ºC and 0.16 M NaCl ionic strength. Potentiometric data were processed using HyperQuad2013 software. Titrations were performed on a 809 Titrando from Metrohm. The temperature was kept constant with a circulating water bath.

Surface plasmon resonance

The interaction of MPI and surface-bound polyP was carried out at 25°C using a T200 Biacore (GE Healthcare Life Sciences). Samples were prepared by making stock

solutions of at least 100 X the desired concentration at high volume and filtered using 0.2 µm filters (Millex, Merck Millipore Ltd). Running buffer was prepared with 20 mM HEPES-NaOH with 100 mM NaCl, 1 mM EDTA and 0.005% P20 surfactant. Running buffer was degassed, filtered and the pH was 7.4.

Prior to titration, stock solutions of MPI were serially diluted with the same running buffer. A multi-cycle assay was run with 1 M NaCl injection after each cycle. Using a flow rate of 30 µL/min and an injection volume of 60 µL, the association injection was run over 120 seconds, whereas the dissociation was run over 60 seconds. 7-8 concentrations for each MPI were run for each steady state affinity plot. All samples were run on a Series S sensor chip CM4 modified with streptavidin (GE Healthcare, Life Sciences) with a biotinylated long chain polyP (P1075), a biotinylated medium chain polyP (P560), a biotinylated platelet sized polyP (P110) and one non-functionalized flow cell for the reference cell. Data were analyzed using the Biacore T200 Software (GE Healthcare, Life Sciences).

Isothermal titration calorimetry

Thermodynamic analyses of the interaction of MPI and polyP were carried out at 25 °C using a MicroCal iTC200 calorimeter (MicroCal, Northampton, USA). Samples were prepared by making stock solutions of at least 100 X the desired concentration at high volume and filtered using 0.2 µm filters (Millex, Merck Millipore Ltd). The pH of each solution was then adjusted to between 7.35 and 7.40. Prior to titration, stock solutions were diluted with same filtered buffer and degassed. Titrations consisted of 25 consecutive injections of 1.5 µL volume and 5 sec duration, with a 3 min interval between injections to return the system heat to baseline. The first sacrificial injection was 0.2 µL in volume and not used as part of the data analysis. Heats of dilution were measured by injecting MPI solution into buffer alone. They were subtracted from the corresponding thermal peaks measured for the sample prior to data analysis. The resulting differential binding heat data were fit into a single set of identical sites model using the MicroCal ORIGIN software supplied with the instrument.

In vitro and in vivo measurements

Determination of polyP inhibition activity by viscosity-based plasma clotting assay

We followed a recent report (Abbina et al Mol. Pharm. 2022, 19 (6), 1853–1865. https://doi.org/10.1021/acs.molpharmaceut.1c00934) for this analysis. MPI solutions were prepared in 10 mM tricine buffer (pH 7.4, 50 μM $ZnCl_2$ and 150 mM NaCl). For inhibition studies, citrated human PPP from Affinity Biologicals (20 donors, pooled) was warmed to 37 °C and incubated with MPI solutions and polyP (700 monomer units at 20 μM final monomer concentration) at 37 °C for 15 minutes such that the final plasma concentration consisted of 50 % of the reaction mixture. The final concentration of MPI in plasma ranged from 2.5 to 100 µg/mL. As a positive control, plasma was incubated with MPI without addition of polyP, keeping the plasma concentration at 50 %. As a negative control, plasma was incubated with tricine buffer, again keeping the same concentration of plasma. Clotting was initiated by addition of a clotting mixture comprised of recombinant tissue factor (Dade® Innovin® rTF, Siemens/Dade-Behring), an 80:20 PCPS mixture and $CaCl_2$ at final concentrations of 0.24 pM, 25 µM and 7.7 mM, respectively. One hundred microliters of the plasma mixture were transferred to cuvette-strips at 37 °C

and clotting was initiated with addition of 50 µL of the clotting mixture. The clotting time was measured on a STart 4® coagulometer (Diagnostica Stago, France). As each experimental cuvette strip had 4 wells, each experiment was run with one negative and one positive controls. All experiments were performed in triplicate and the average values (mean ± standard error of the mean) are reported.

Influence of fibrin clot structure and fiber diameter by scanning electron microscopy (SEM)

The effects of MPI 8 on fibrin clot structure and fibrin diameter in the presence of MPI 8 were assessed by SEM. All samples were randomly coded and blinded to the individual performing the imaging analysis to avoid bias. Fibrin clots were prepared in sterile, 5 mL round-bottom polypropylene tubes (BD Falcon) by mixing human fibrinogen (2.6 mg/mL) in 20 mM HEPES (pH 7.4 and 150 mM NaCl) buffer with 2.5 mM $CaCl_2$ (final). When indicated, MPI 8 (20 µg/mL) or polyP (P700, 100 µM) were added. Control clots were prepared in the absence of MPI 8 or polyP. Clotting was initiated with 3 nM thrombin. Clots were then allowed to mature for 1 hour and processed for SEM imaging.

The clot samples were rinsed three times using HEPES buffer then fixed with Karnovsky fixative (2.5% glutaraldehyde and 4% formaldehyde). To allow for better penetration of the buffer solutions into the clot, a PELCO344I Laboratory Microwave System was used between buffer changes. After clot fixation, the clot sample was washed three times using fresh 0.1 M sodium cacodylate buffer before staining using 1% osmium tetraoxide dissolved in 0.1 M sodium cacodylate buffer in the microwave. The clot sample was washed gently using distilled water for a minimum of five exchanges and resuspended in 50% ethanol solution. It was left to incubate for 10 minutes at room temperature before subjecting it to the microwave. The dehydration procedure was repeated using a graded series of ethanol solutions (70%, 80%, 90%, and 95% ethanol in water followed by 100% ethanol three times). Once the sample was fully dehydrated, it was placed in a Tousimis Autosamdri 815B Critical Point Dryer overnight under stasis mode before fully completing the drying process the following day. Finally, the processed samples were mounted on SEM stubs using hot glue and coated in 10 nm of Au/Pd coating (16.38 g/cm^3) using a Cressington 208HR High Resolution Sputter Coater. Samples were stored in a desiccator. Clot images were captured on a Helios NanoLab 650 SEM at different magnifications (5000X, 10000X and 25000X). Multiple images from different areas of each clot were captured. Fiber diameters of clots were measured with ImageJ. For fibrin fiber diameter calculations, images from two independent experiments were analysed. Fibrin fiber diameters (n = 80) from 4 separate areas of each clot were used to calculate the mean fiber diameter.

Platelet activation by ADP in presence of MPI 8

Whole blood collected at 1:9 into 3.8% sodium citrate tubes was centrifuged at 156 g for 12 min to generate platelet rich plasma (PRP). The influence of MPI 8 on ADP-mediated platelet activation was assessed by pre-mixing the MPI 8 with ADP (40 µM final) (10 µL total) immediately before the addition of 90 µL of PRP, and the resulting suspension was incubated for 15 min at 37 ˚C. Controls containing PBS alone (vehicle) as well as ADP without MPI-8 were also included. Activation was then assessed by flow cytometry (CytoFLEX Flow Cytometer, Beckman Coulter). Briefly, 5 µL of PRP suspension was

added to 50 µL of PBS containing 20X-diluted anti-human CD62P-PE (BD Biosciences, PE Mouse Anti-Human CD62P, BD Biosciences, cat. no. 550561 (lot 9037907), clone AC1.2, dilution: 1 in 22). Platelets were gated based on anti-human CD42-FITC (Anti-CD42a-FITC: FITC Mouse Anti-Human CD42a from BD Biosciences, cat. no. 558818 (lot 9108735), clone ALMA.16, dilution: 1 in 22), prepared in the same manner. Using this gate, 10,000 events were counted, and activation was quantified by the percentage of cells positive for CD62P.

Inhibition of nucleic acid mediated initiation of coagulation by MPIs

MPIs at two concentrations were incubated with pooled poor platelet plasma 2-fold diluted with HEPES buffer. The plasma MPI mixture was spiked with 67.11 µg/mL of Low molecular weight Polyinosinic:Polycytidylic acid (poly I:C) (surrogate for nucleic acids) for 15 minutes. 100µL this mixture was transferred to a cuvette strip with magnetic balls and incubated for 3 min at 37 ^{o}C on Stago Start 4 coagulometer. Clotting was triggered by 50 µL of trigger reagent containing 25 mM $CaCl_2$, diluted 1:15,000 re-lipidated tissue factor (Dade Innovin) and 1mM PCPS lipid vesicles. Clotting time is calculated between the time of addition of trigger reagent and when the magnetic bead stops oscillating.

Inhibition of thrombosis by MPI via $FeCl_3$ induced injury to carotid arteries

The animal protocol was approved by the University of Michigan. Male and female eight to ten week old C57/BL6 mice were obtained from The Jackson Laboratories (Bar Harbor, ME). Light cycles in the animal holding rooms are set for 12 hours on and 12 hours off. Temperature, humidity and airflow are maintained and controlled. Mice are caged in autoclaved ventilated caging at a capacity of 4 animals/cage during the course of the experiment. Mice were anesthetized by using an inhaled isoflurane-oxygen mixture. MPI and UHRA compounds diluted in sterile normal saline were injected retro-orbitally. The left carotid artery was exposed via a midline cervical incision and blunt dissection, and blood flow was monitored with a Doppler vascular flow probe (Transonic 0.5PSB) connected to a perivascular flowmeter (Transonic TS420). To induce thrombosis, two 1 X 2-mm pieces of filter paper (Whatman GB003) saturated with freshly prepared 7.5 % anhydrous $FeCl_3$ in 0.9% saline were applied to the deep and superficial surfaces of the artery. After 3 minutes, the filter papers were removed, and the vessel was irrigated with warm saline. Blood flow was monitored from $FeCl_3$ application for 30 minutes or until occlusion, defined as no detectable flow for 3 minutes. Mice were then euthanized by cervical dislocation while still under anesthesia. Flow data were interpreted with LabScribe2 (iWorx Systems). Data for this study are reported as mean (n = 8 mice) ± standard deviation.

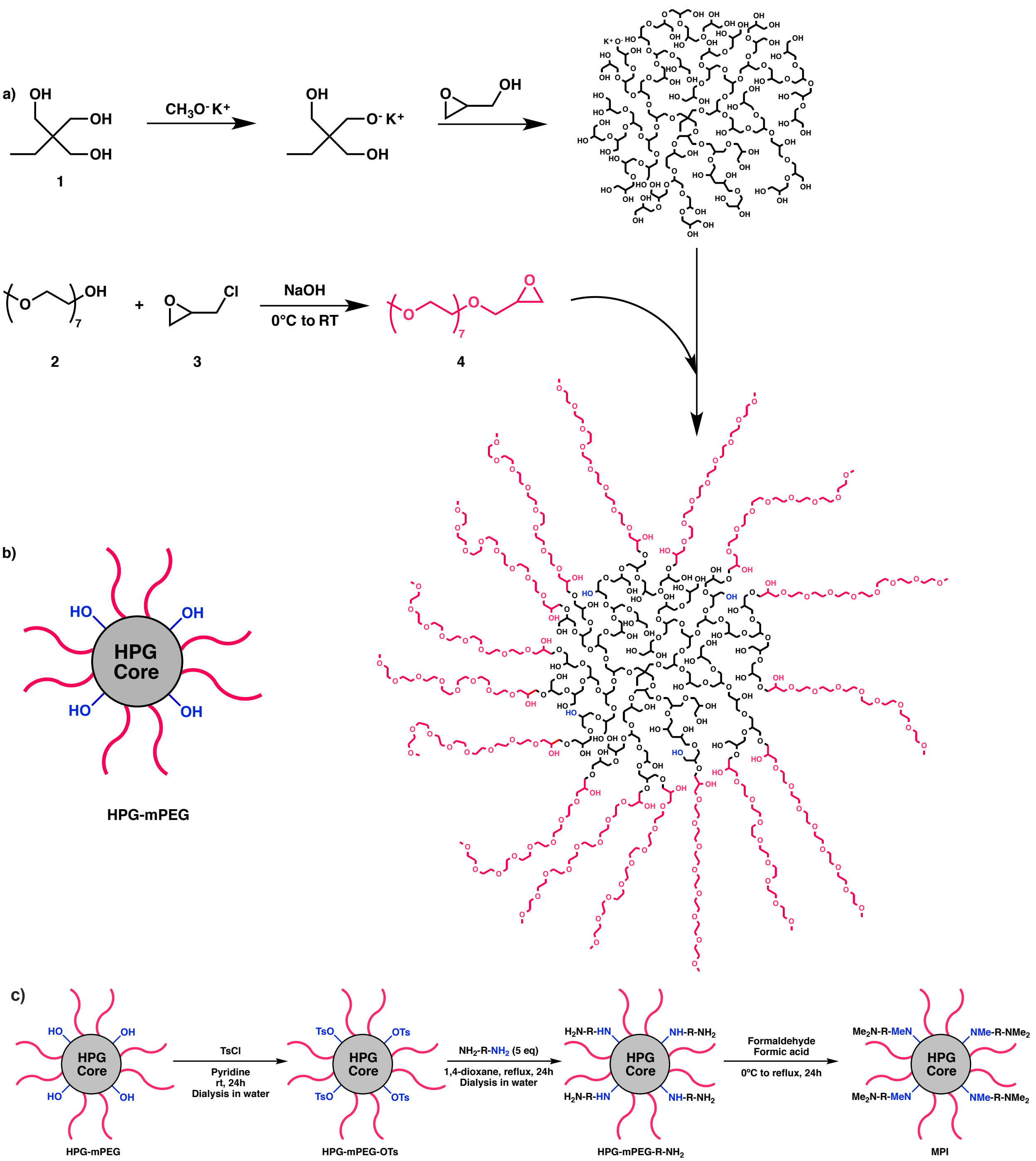

Supplementary Figure 1. Synthetic scheme for polymer scaffold, HPG-mPEG$_{35}$ and post-polymerization modification. a) Anionic ring opening polymerization of glycidol to form HPG, followed by in situ addition of mPEG$_{350}$ epoxide. **b)** Simplified representation of polymer scaffold, HPG- mPEG$_{350}$. **c)** Post polymerization modification of HPG-mPEG$_{350}$.

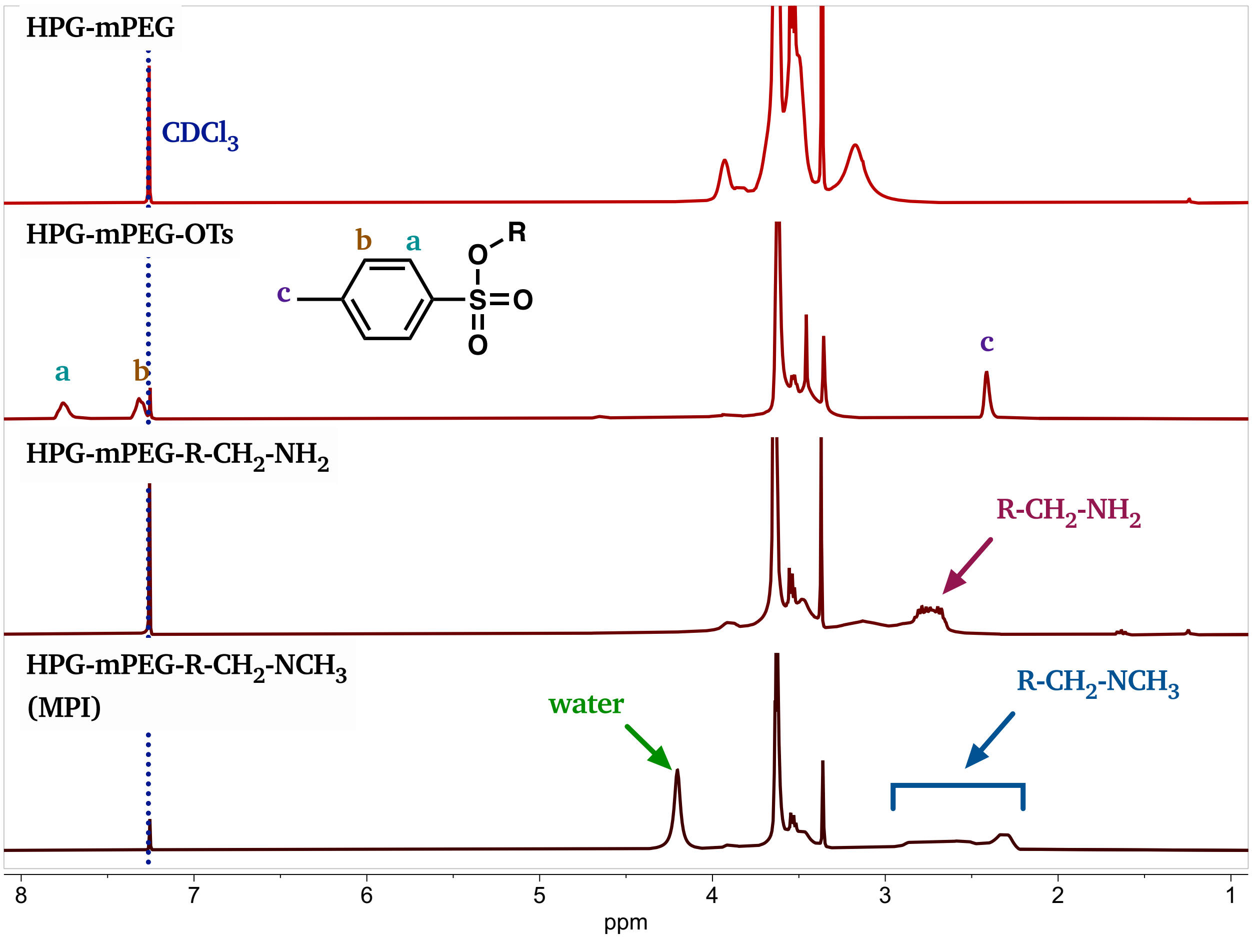

Supplementary Figure 2. ^{1}**H NMR taken on 300 MHz spectrometer in $CDCl_3$ of HPG-mPEG$_{350}$. The spectra were acquired at 22 ˚C.**

From top to bottom, each step of the post polymerization process of HPG-mPEG to MPI is shown.

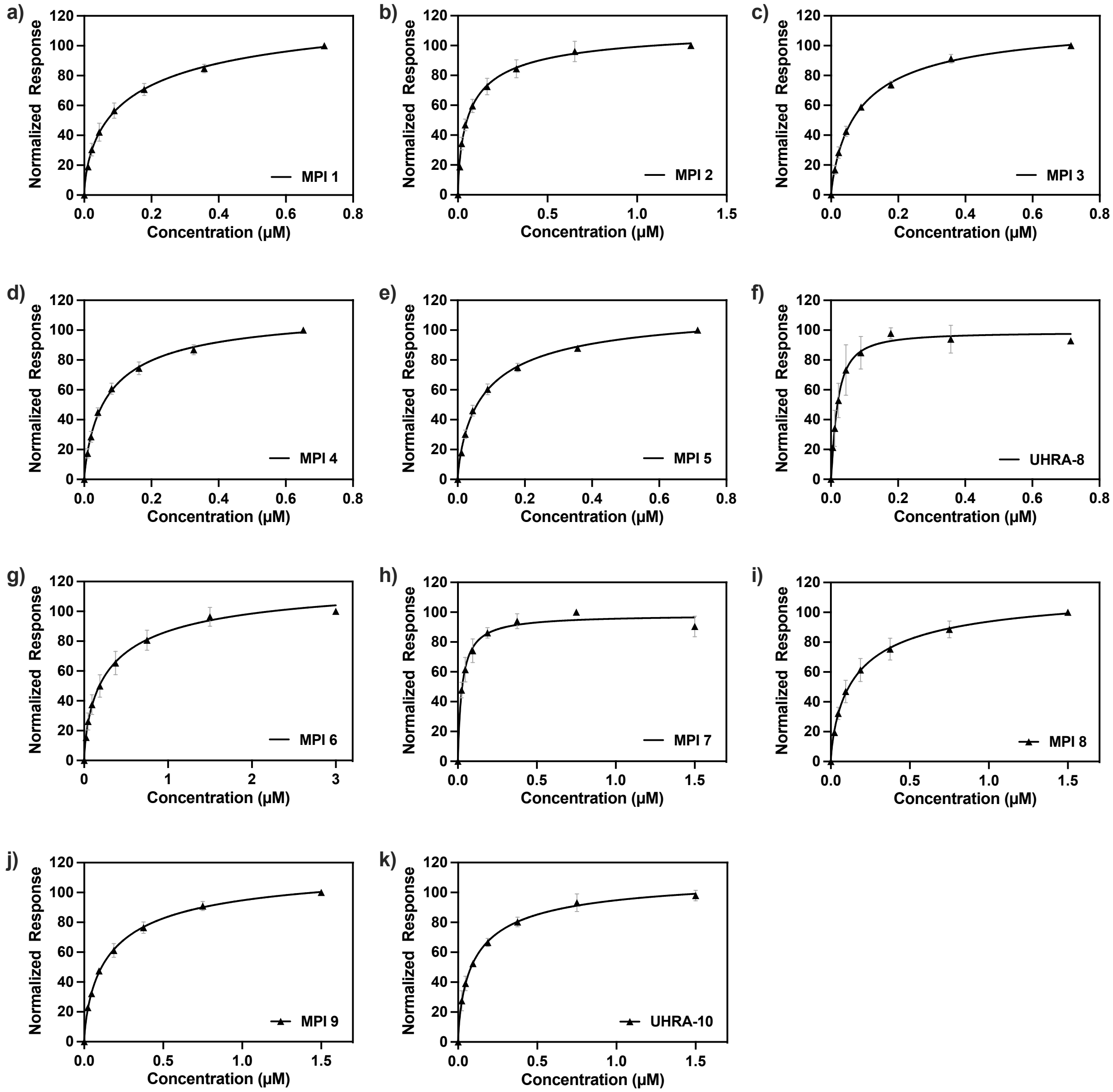

Supplementary Figure 3. Summary binding curves for each MPI binding to SC polyP (P110) using surface plasmon resonance (SPR).

Binding affinities were obtained from the steady state affinity for each MPI. All concentrations were run at 25 ºC in HBS running buffer with EDTA and P20 surfactant. All results represent mean values +/- SD of n = 3 independent replicates. a)-e) represent MPI 1-5. f) UHRA-8. g)-j) MPI 6-9. k) UHRA-10.

A.

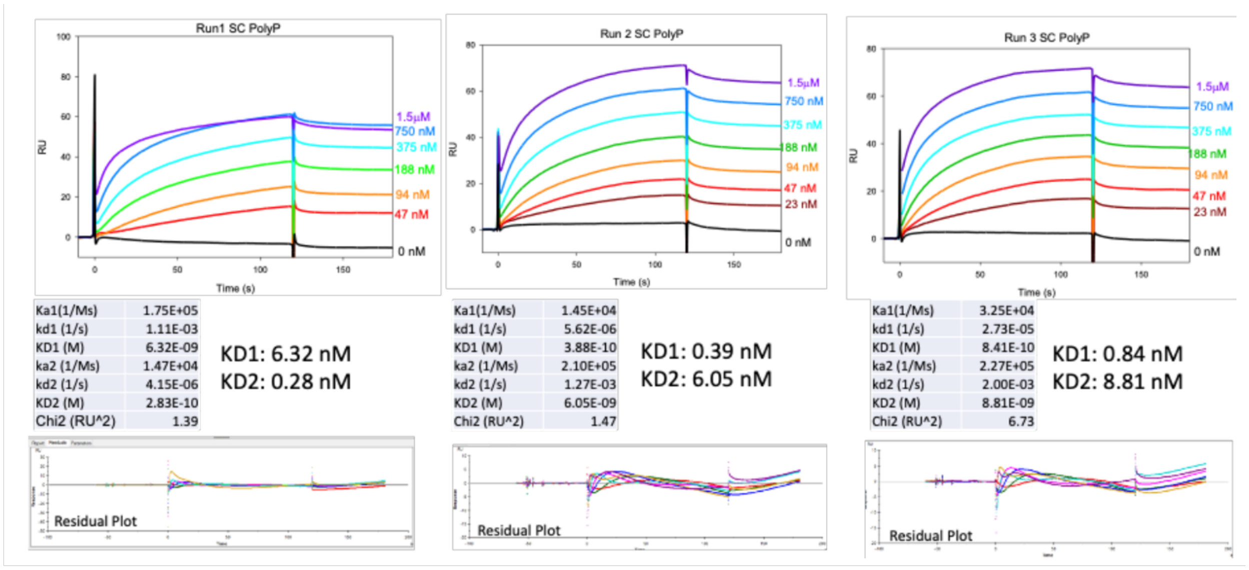

B.

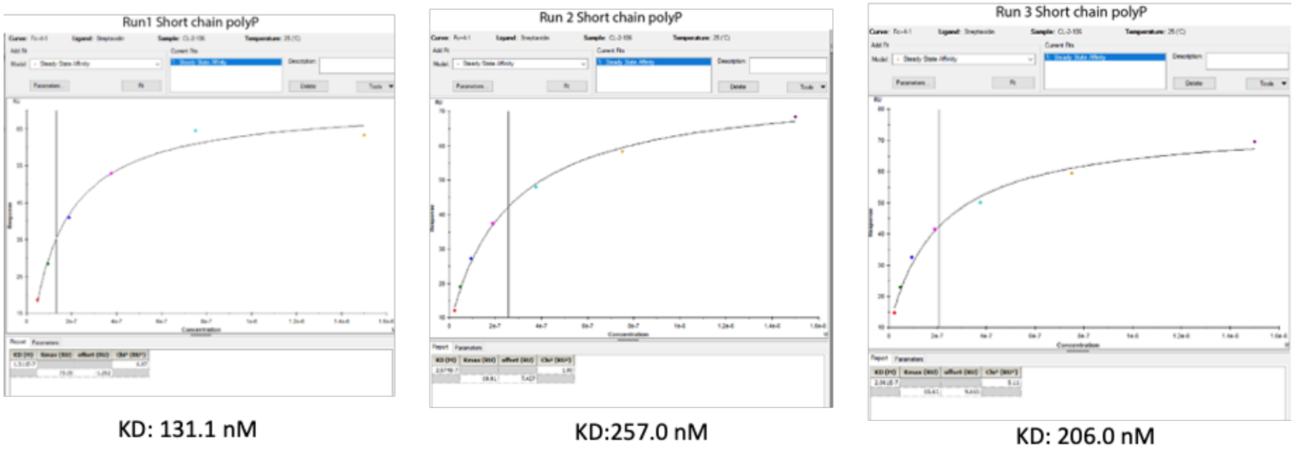

Supplementary Figure 4: Representative row SPR binding curves for MPI-8 with short chain polyP. **A)** SPR curves, binding constant and residual plots for short chain polyP binding to MPI 8 collected by SPR analyses are show for three independent runs. Heterogenous ligand curve fitting is shown. **B**) Affinity plots for short chain polyP with MPI 8 from Biacore Software. Mean KD= 198 ± 36 nM.

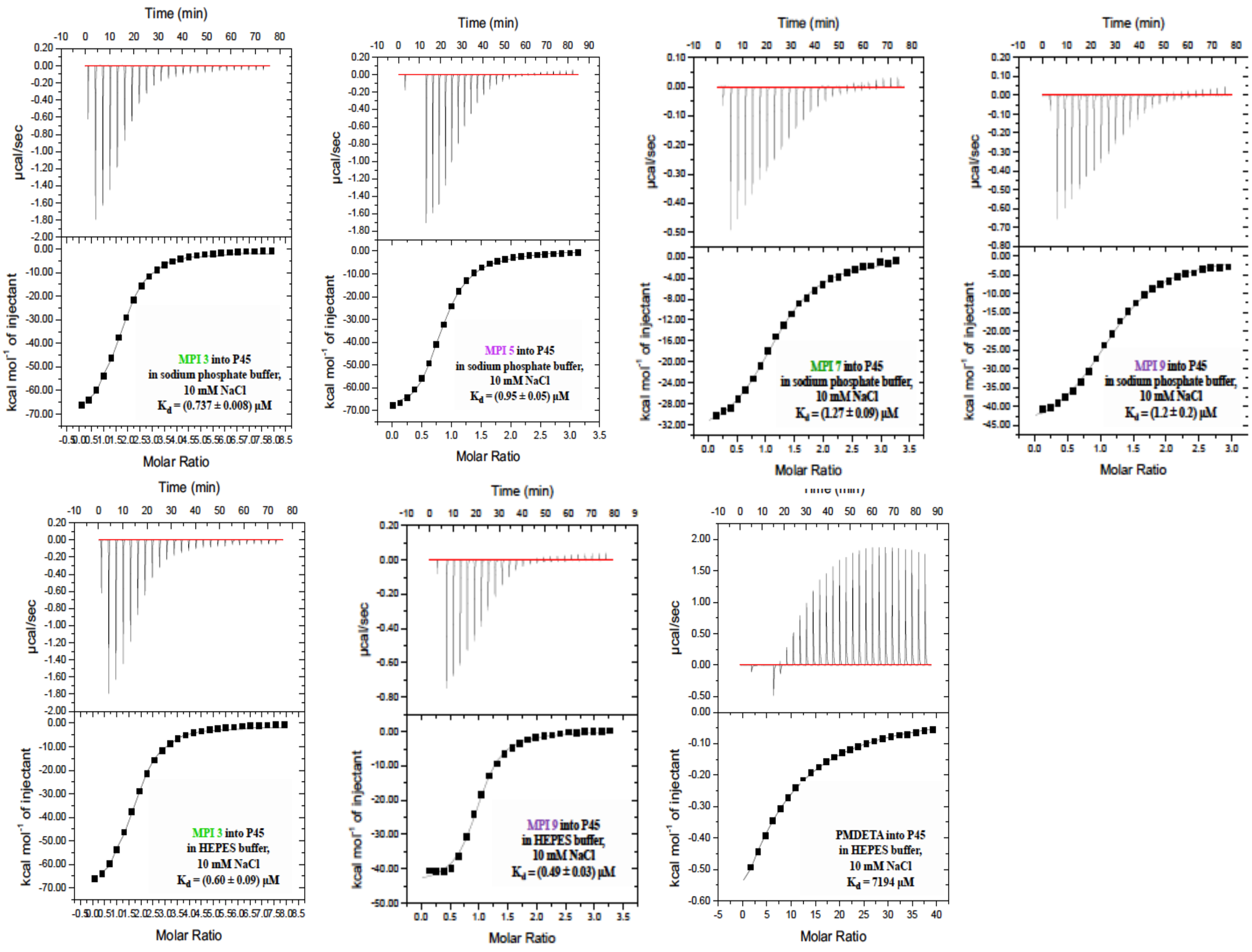

Supplementary Figure 5: Thermograms and differential binding curves obtained by ITC for select MPI binding to polyP with 10 mM NaCl added, as labelled. All experiments were conducted at pH 7.4 and 25 °C. One representative titration is shown for each system studied, while the thermodynamic data reported were taken as the mean of three independent titrations, each normalized by their respective heats of dilution.

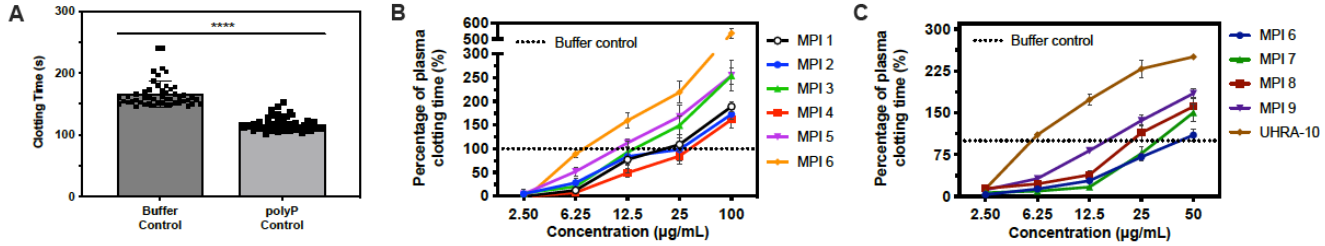

Supplementary Figure 6. Inhibition of polyP procoagulant effects on plasma clot times.

Percentage of plasma clotting time calculated from clot time of buffer control (100 %) and polyP control (0 %). Plasma clotting assay performed using human PPP, 20 donors pooled. Negative control: PPP with tricine buffer. Positive control: PPP with polyP (700 monomer units, 20 µM monomer concentration). **a)** Actual clot time of positive and negative controls, showing a significant decrease in clot time upon addition of polyP. Data were statistically significantly different at $p < 0.0001$ in an unpaired, two-tailed T-test with Welch's correction. **b)-c)** Calculated percentages of clotting time to demonstrate polyP inhibition. The dotted line indicates the value for plasma incubated with buffer (*i.e.*, buffer control). All results represent mean values +/- SD of n = 3 independent replicates.

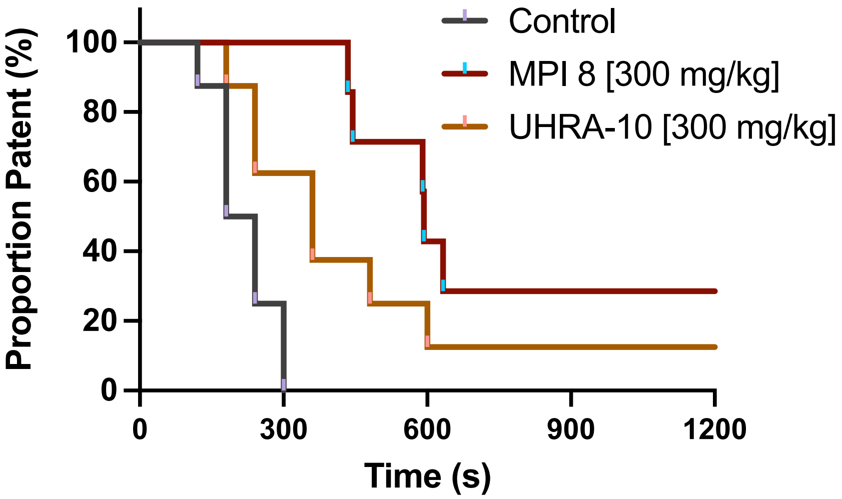

Supplementary Figure 7. MPI 8 delays time to occlusion in carotid artery thrombosis model. Artery patency was monitored by Doppler flow probe. Injury was induced by topical application of $FeCl_3$ and patency is plotted versus time, comparing the saline control, MPI 8 and UHRA-10. At 300 mg/kg, MPI 8 and UHRA-10 have reached a similar level of patency, likely a maximum in this model by these inhibitors. Results shown are mean of n = 8 mice. A log rank analysis tests indicate the two curves are significantly different (***P<0.0005).

A.

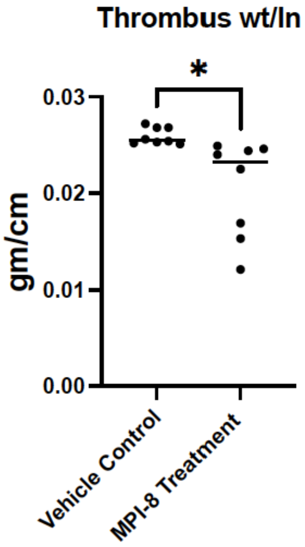

B.

Table Analyzed	Thrombus wt/ln
Column B	MPI-8 Treatment
vs.	vs.
Column A	Vehicle Control
Unpaired t test with Welch's correction	
P value	0.0201
P value summary	*
Significantly different (P < 0.05)?	Yes
One- or two-tailed P value?	Two-tailed
Welch-corrected t, df	t=2.950, df=7.403
How big is the difference?	
Mean of column A	0.02593
Mean of column B	0.02059
Difference between means (B - A) ± SEM	-0.005338 ± 0.001809
95% confidence interval	-0.009568 to -0.001107
R squared (eta squared)	0.5404
F test to compare variances	
F, DFn, Dfd	34.69, 7, 7
P value	0.0001
P value summary	***
Significantly different (P < 0.05)?	Yes
Data analyzed	
Sample size, column A	8
Sample size, column B	8

Supplementary Figure 8. Inferior vena cava stenosis thrombosis model. A. MPI 8 treat yielded significantly smaller thrombus than control treatment as measured by thrombus weight/length (p=0.0201). **B.** Parameters used for statistical analysis is shown. Approximately 18 - 24 hours after MPI 8 was delivered, an IVC stenosis (partial flow) ligation as described (Ref. PMID 30786739 and PMID 26482993) was performed to induce thrombosis. Thrombosis was achieved in 8 of 10 mice for each group (at 48 hours post-VT), and these 8 values were used for comparison. Student' t-Test was used for statistical comparison.

Table Analyzed	Thrombus weight
Column B	MPI-8 Treatment
vs.	vs.
Column A	Vehicle Control
Unpaired t test with Welch's correction	
P value	0.0014
P value summary	**
Significantly different (P < 0.05)?	Yes
One- or two-tailed P value?	Two-tailed
Welch-corrected t, df	t=4.791, df=8.032
How big is the difference?	
Mean of column A	0.02330
Mean of column B	0.01245
Difference between means (B - A) ± SEM	-0.01085 ± 0.002265
95% confidence interval	-0.01607 to -0.005631
R squared (eta squared)	0.7408
F test to compare variances	
F, DFn, Dfd	13.49, 7, 7
P value	0.0028
P value summary	**
Significantly different (P < 0.05)?	Yes
Data analyzed	
Sample size, column A	8
Sample size, column B	8

Supplementary Figure 9. Inferior vena cava stenosis thrombosis model. MPI 8 treat yielded significantly smaller thrombus than control treatment as measured by thrombus weight (p=0.0014) (please refer to **Figure 5 I** for the data. **The parameters used for statistical analysis for the data given in figure 5 I is shown.** Approximately 18 - 24 hours after MPI 8 was delivered, an IVC stenosis (partial flow) ligation as described (Ref. PMID 30786739 and PMID 26482993) was performed to induce thrombosis. Thrombosis was achieved in 8 of 10 mice for each group (at 48 hours post-VT), and these 8 values were used for comparison. Student' t-Test was used for statistical comparison.

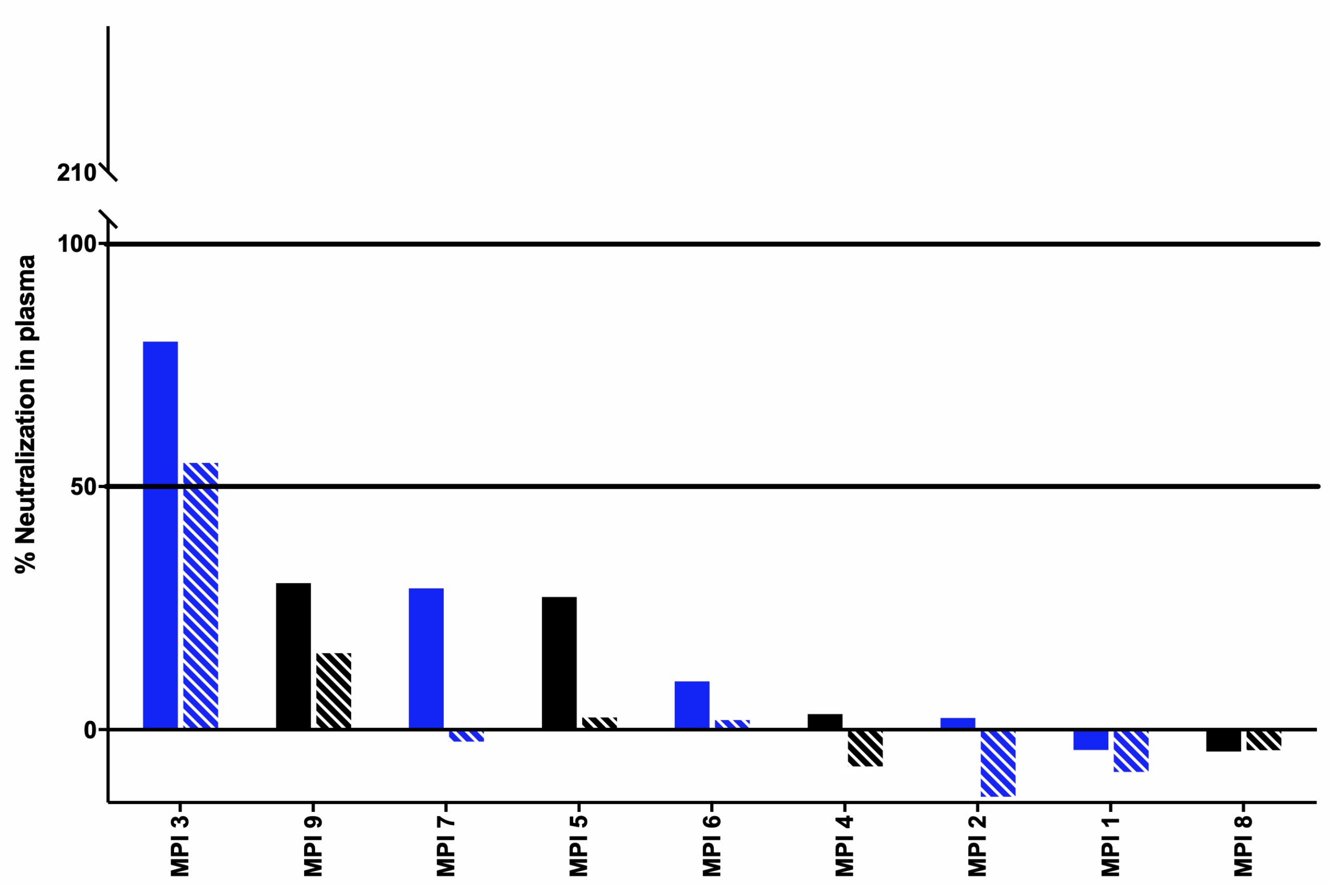

Supplementary Figure 10: Inhibition of activation of blood coagulation by nucleic acid (surrogate nucleic acid Poly IC) by MPIs. Screening of inhibitors using plasma clotting assay to measure blood coagulation initiation. The data is represented as percent neutralization with 0% being the Poly IC with no inhibitor and 100% being the plasma with no poly IC and no inhibitor. The solid bars indicate 200 µg/mL and striped bar indicate 100 µg/mL of inhibitor concentration. MPI-8 did not inhibit PolyIC initiated blood coagulation.

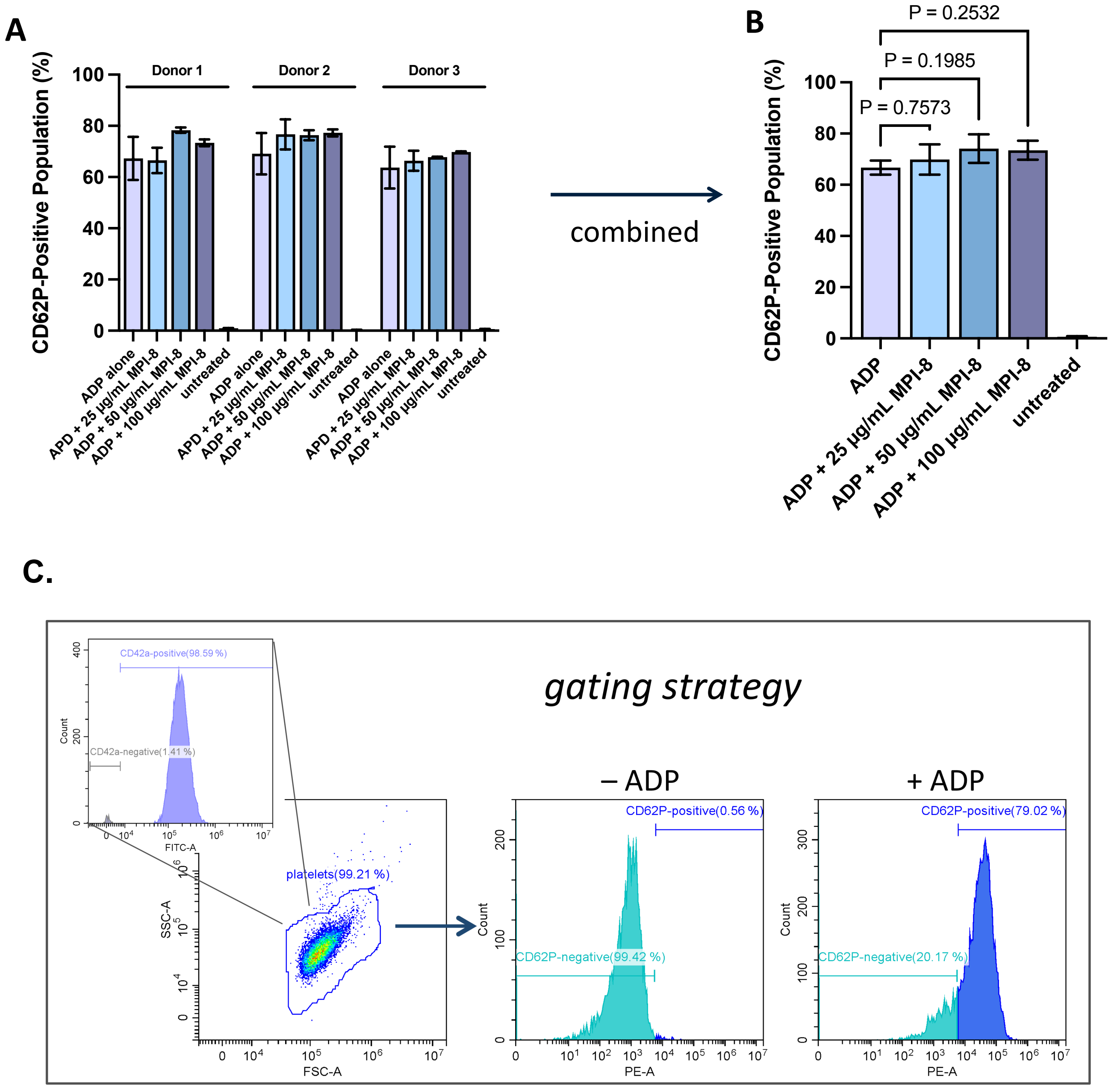

Supplementary Figure 11: Platelet activation when ADP and MPI-8 are added together in platelet rich plasma using flow cytometry. A) Platelet activation data from three independent human donors, **B)** Combined data from the analysis, **C)** Illustration of the gating strategy used for flow cytometry analysis. MPI-8 was added at different concentrations to ADP and incubated with platelet rich plasma (PRP). Platelet activation was measured using flow cytometry by measuring the expression of activation marker CD62P using mouse anti-human CD62P-PE antibody (cat. no. 550561, BD Biosciences). No platelet activation was observed when MPI-8 alone was incubated with PRP at any concentration. Data is reported as mean ± standard deviation. Statistical analysis was conducted using one-way ANOVA (P = 0.3791) with Dunnett's multiple comparisons test (reported in figure).

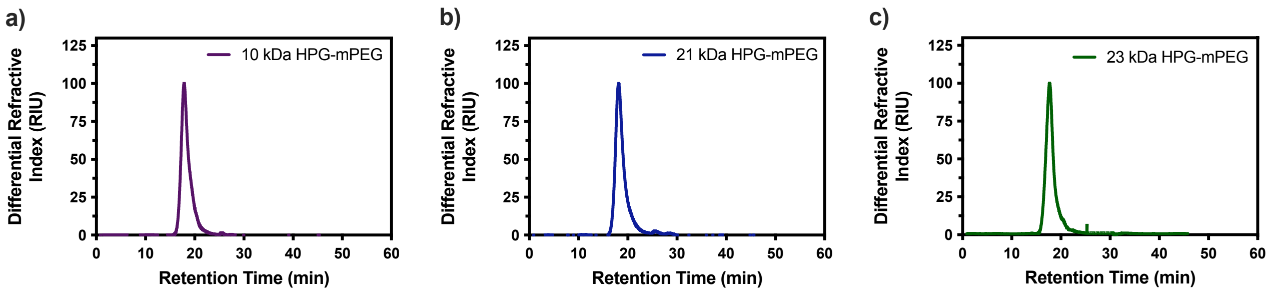

Supplementary Figure 12. GPC chromatograms for the unfunctionalized HPG-mPEG scaffolds used to build the MPI library. **a)** 10 kDa scaffold. **b)** 21 kDa scaffold. **c)** 23 kDa scaffold.

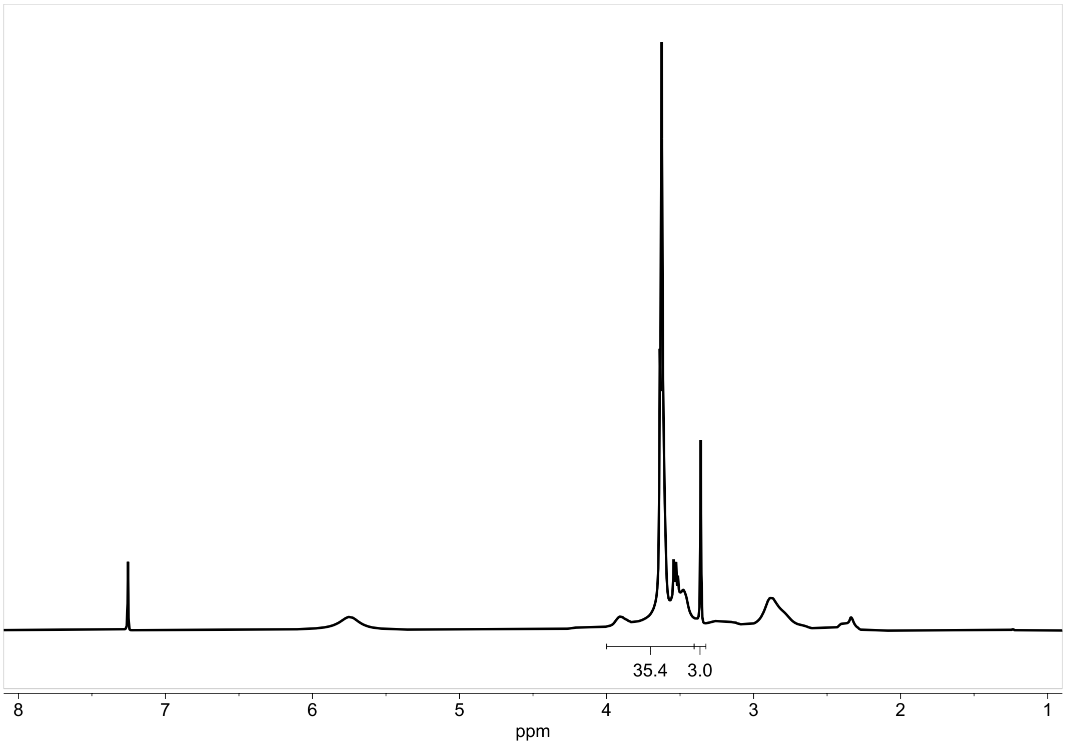

Supplementary Figure 13. ^{1}H NMR of MPI 1 in $CDCl_3$. **^{1}H NMR taken on 300 MHz spectrometer in $CDCl_3$. The spectrum was acquired at 22 ˚C.**

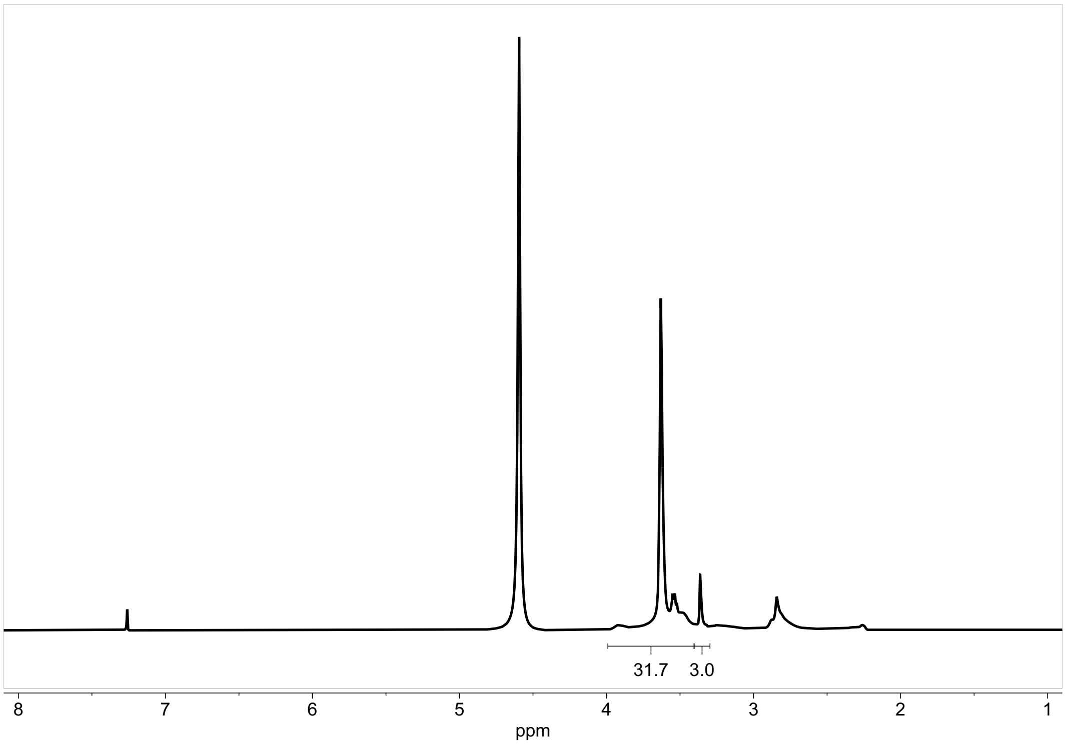

Supplementary Figure 14. ^{1}H NMR of MPI 2 in $CDCl_3$. **^{1}H NMR taken on 300 MHz spectrometer in $CDCl_3$. The spectrum was acquired at 22 °C.**

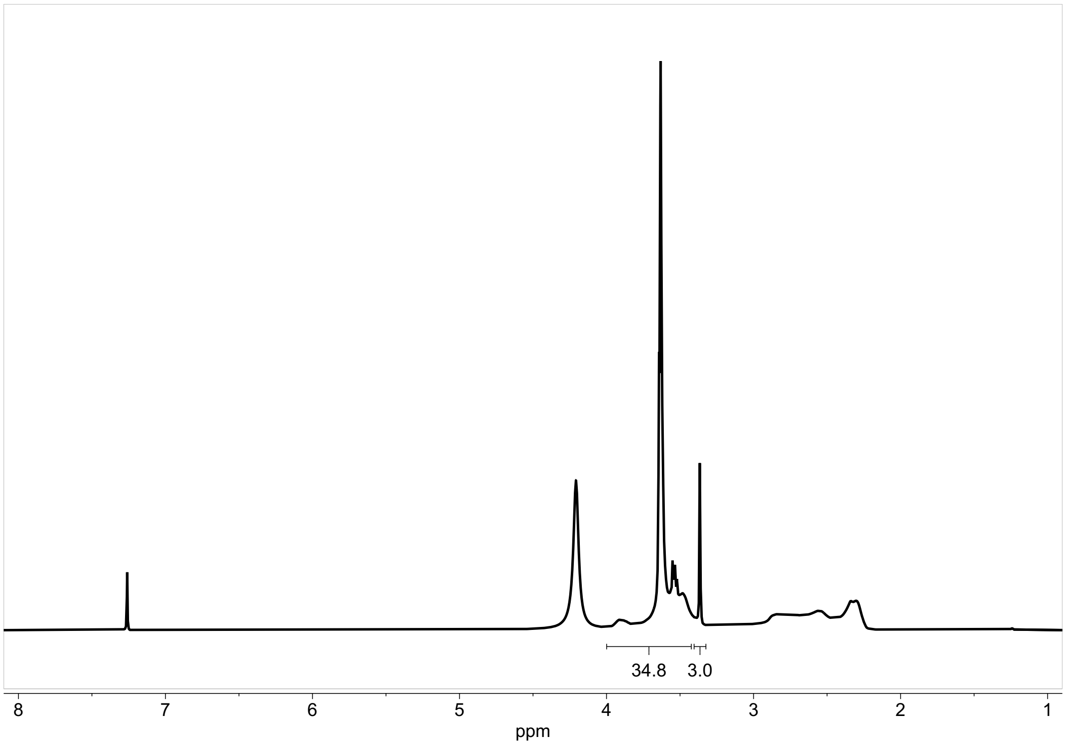

Supplementary Figure 15. ^{1}H NMR of MPI 3 in $CDCl_3$. **^{1}H NMR taken on 300 MHz spectrometer in $CDCl_3$. The spectrum was acquired at 22 ˚C.**

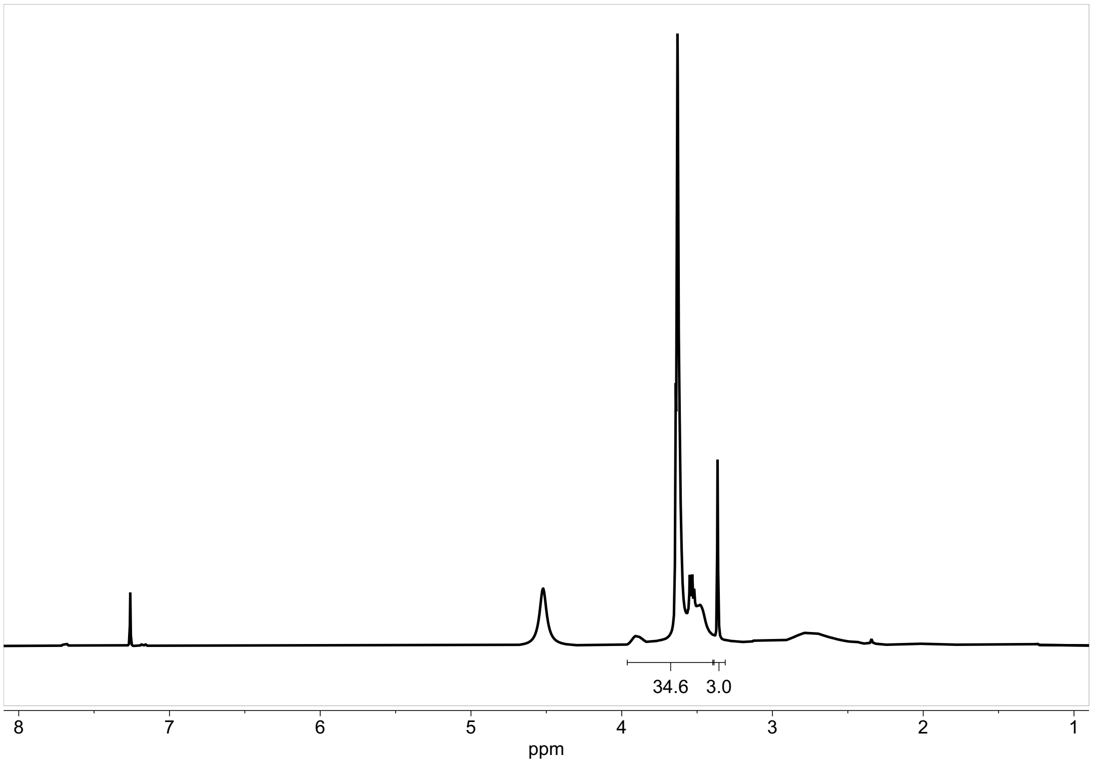

Supplementary Figure 16. ^{1}H NMR of MPI 4 in $CDCl_3$. **^{1}H NMR taken on 300 MHz spectrometer in $CDCl_3$. The spectrum was acquired at 22 ˚C.**

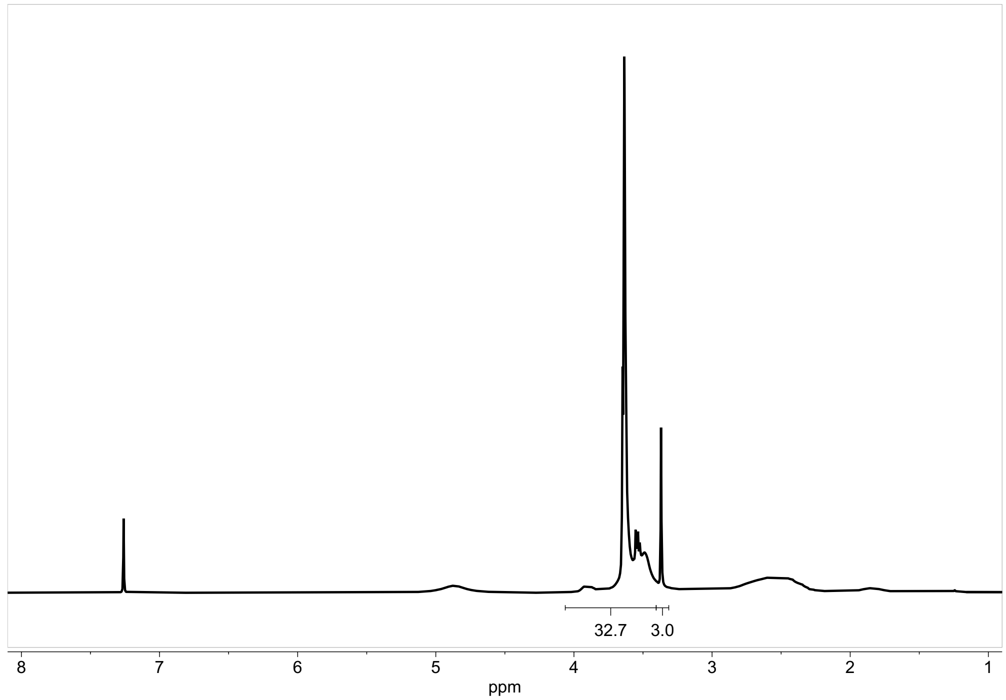

Supplementary Figure 17. ^{1}H NMR of MPI 5 in $CDCl_3$. **^{1}H NMR taken on 300 MHz spectrometer in $CDCl_3$. The spectrum was acquired at 22 ˚C.**

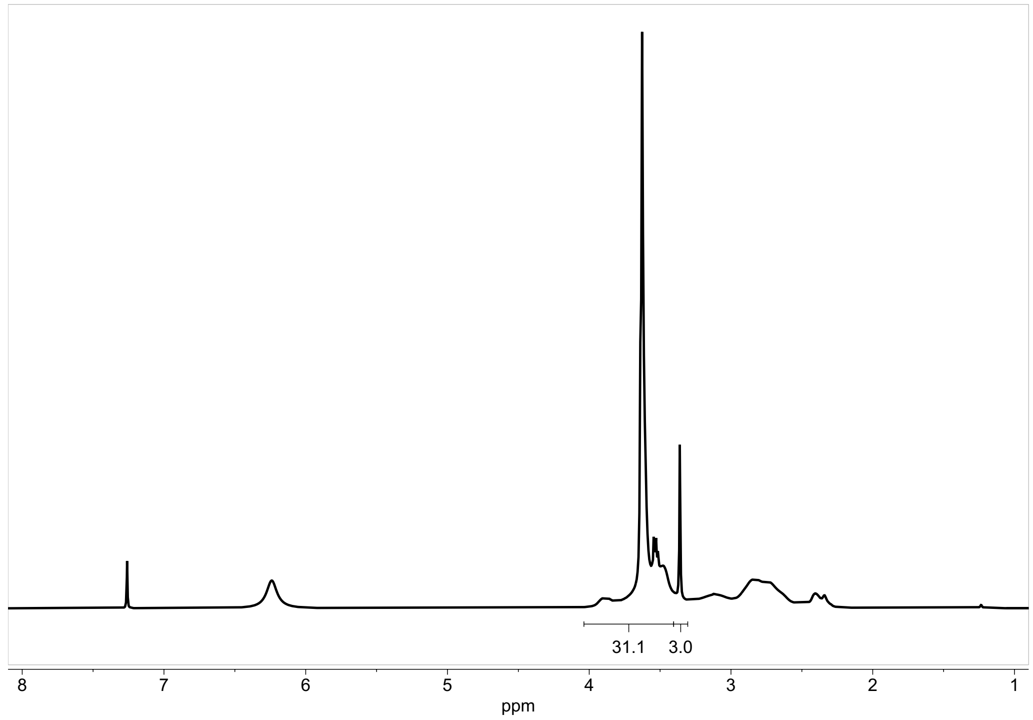

Supplementary Figure 18. ^{1}H NMR of MPI 6 in $CDCl_3$. **^{1}H NMR taken on 300 MHz spectrometer in $CDCl_3$. The spectrum was acquired at 22 ˚C.**

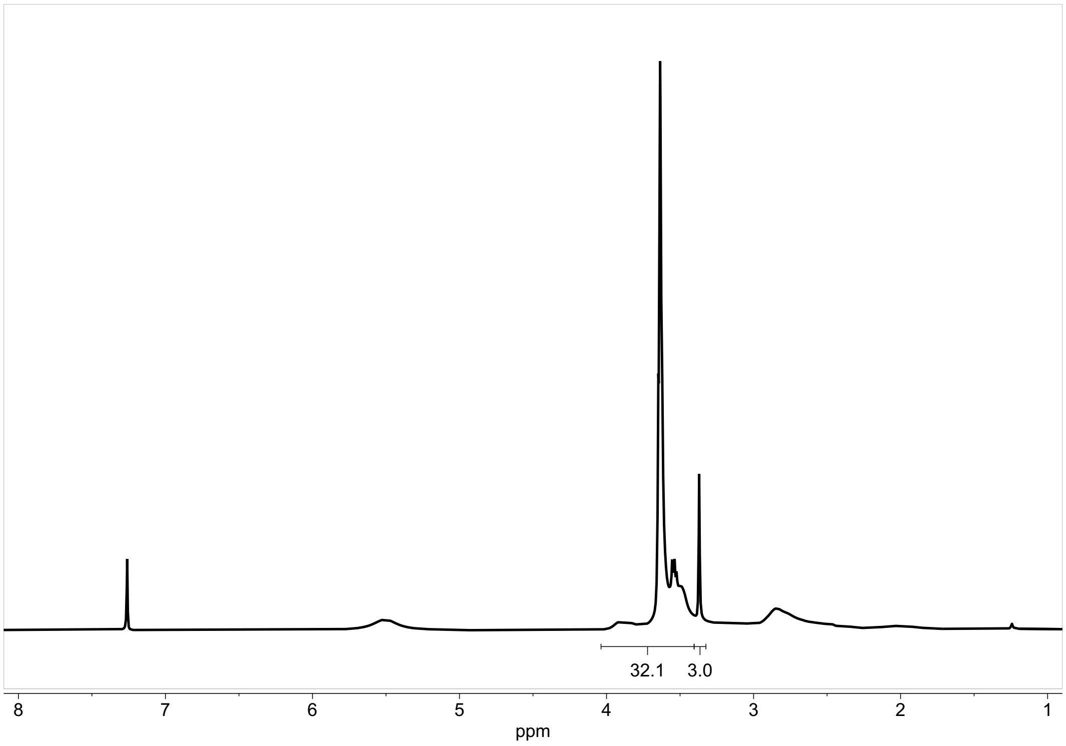

Supplementary Figure 19. ^{1}H NMR of MPI 8 in $CDCl_3$. **^{1}H NMR taken on 300 MHz spectrometer in $CDCl_3$. The spectrum was acquired at 22 ˚C.**

Supplementary Table 1. General structures of selected representative amine structures analyzed for protonation state behavior in the design of novel CBG.

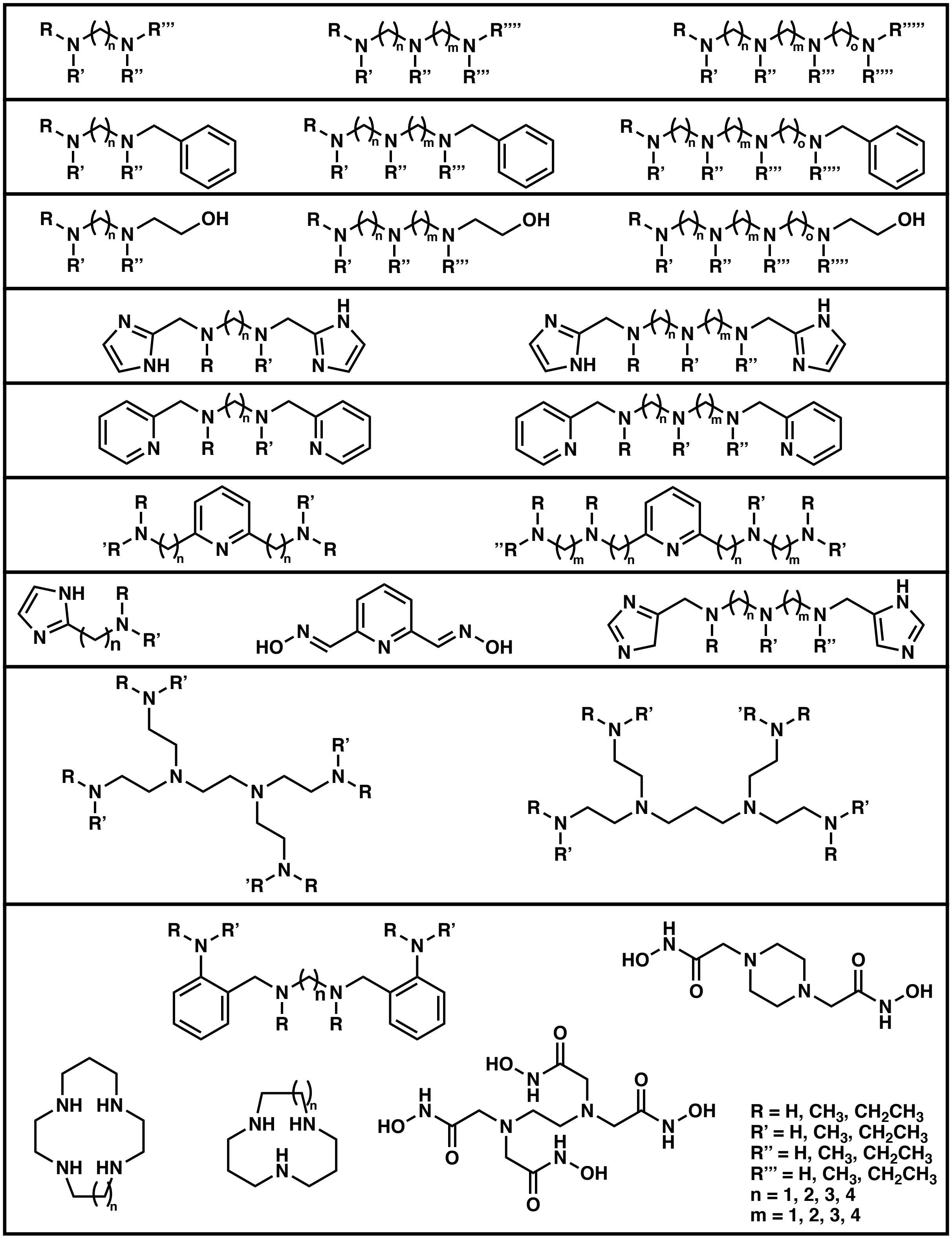

Supplementary Table 2. Summary of log K values obtained from potentiometry for CBGs loaded on MPIs, before and after conjugation.

	$L + H \rightleftharpoons HL$	$HL + H \rightleftharpoons H_2L$	$H_2L + H \rightleftharpoons H_3L$	$H_3L + H \rightleftharpoons H_4L$
**CBG I**	9.22 ± 0.02	8.41 ± 0.02	2.09 ± 0.02	-
**MPI 2**	8.7 ± 0.4	7.5 ± 0.2	4.3 ± 0.1	-
**CBG II**	10.6 ± 0.2	8.3 ± 0.2	4.1 ± 0.1	-
**MPI 4**	8.9 ± 0.2	6.5 ± 0.3	3.6 ± 0.1	-
**Me_6-TREN**	9.50	8.68	7.44	< 1.0
**UHRA-8**	9.2 ± 0.2	7.5 ± 0.1	4.1 ± 0.1	< 1.0

Potential in mV was measured as standardized base (0.15 M NaOH) and titrated into acidified (pH 2) solution of CBG or MPI at 25 ºC, 160 mM NaCl. Relatively similar values were obtained for other MPIs.

Supplementary Table 3. Summary of binding thermodynamics, stoichiometry and binding affinity data obtained by isothermal titration calorimetry (ITC) at pH 7.4 and 25 °C.

Buffer	NaCl (mM)	Cell (titrand)	Syringe (titrant)	N	K_d (µM)	∆G (kcal/mol)	∆H (kcal/mol)	T∆S (kcal/mol)
HEPES[c]	10	P45	CBG I	5.1	7194	-2.92	-2.06	0.87
HEPES[c]	10	P45	MPI 9	1.1	0.49 ± 0.03	-8.66 ± 0.04	-37.9 ± 0.9	-29.2 ± 0.9
HEPES[c]	10	P45	MPI 3	0.7	0.60 ± 0.09	-8.50 ± 0.08	-74.8 ± 0.8	-66.3 ± 0.4

[c] HEPES buffer consisted of HEPES (4-(2-hydroxyethyl)-1-piperazineethanesulfonic acid) and NaCl.

Supplementary Table 4. Half maximal inhibitory concentrations of MPI towards long chain and short chain polyP via the thrombin generation assay.

	IC_{50} Long chain polyP (µg/mL)	IC_{50} Short chain polyP (µg/mL)
**MPI 1**	6.6 ± 0.2	1.9 ± 0.1
**MPI 2**	11.5 ± 1.5	2.8 ± 0.4
**MPI 3**	3.8 ± 0.5	1.7 ± 0.3
**MPI 4**	14.3 ± 5.3	2.9 ± 0.5
**MPI 5**	6.0 ± 2.0	1.6 ± 0.2
**UHRA-8**	N/A	1.9 ± 0.5
**MPI 6**	5.3 ± 2.1	1.7 ± 0.4
**MPI 7**	5.6 ± 2.3	1.3 ± 0.1
**MPI 8**	8.5 ± 2.9	1.8 ± 0.2
**MPI 9**	7.6 ± 2.4	0.9 ± 0.1
**UHRA-10**	11.9 ± 10.1	1.4 ± 0.1
