# Supplementary material for: Smart thrombosis inhibitors without bleeding side effects via charge tunable ligand design
Source: Nat Commun. 2023 Apr 26;14:2177. doi: 10.1038/s41467-023-37709-0 (PMC10133246; doi:10.1038/s41467-023-37709-0)
Supplement: Supplementary file 3 — Reporting Summary [file 41467_2023_37709_MOESM3_ESM.pdf]

nature portfolio

Corresponding author(s): J.N. Kizhakkedathu & J.H. Morrissey

Last updated by author(s): Mar 20, 2023

Reporting Summary

Nature Portfolio wishes to improve the reproducibility of the work that we publish. This form provides structure for consistency and transparency in reporting. For further information on Nature Portfolio policies, see our Editorial Policies and the Editorial Policy Checklist.

Statistics

For all statistical analyses, confirm that the following items are present in the figure legend, table legend, main text, or Methods section.

n/a	Confirmed	
☐	☒	The exact sample size (*n*) for each experimental group/condition, given as a discrete number and unit of measurement
☐	☒	A statement on whether measurements were taken from distinct samples or whether the same sample was measured repeatedly
☐	☒	The statistical test(s) used AND whether they are one- or two-sided<br>*Only common tests should be described solely by name; describe more complex techniques in the Methods section.*
☐	☒	A description of all covariates tested
☒	☐	A description of any assumptions or corrections, such as tests of normality and adjustment for multiple comparisons
☐	☒	A full description of the statistical parameters including central tendency (e.g. means) or other basic estimates (e.g. regression coefficient) AND variation (e.g. standard deviation) or associated estimates of uncertainty (e.g. confidence intervals)
☐	☒	For null hypothesis testing, the test statistic (e.g. *F*, *t*, *r*) with confidence intervals, effect sizes, degrees of freedom and *P* value noted<br>*Give P values as exact values whenever suitable.*
☒	☐	For Bayesian analysis, information on the choice of priors and Markov chain Monte Carlo settings
☒	☐	For hierarchical and complex designs, identification of the appropriate level for tests and full reporting of outcomes
☒	☐	Estimates of effect sizes (e.g. Cohen's *d*, Pearson's *r*), indicating how they were calculated

Our web collection on statistics for biologists contains articles on many of the points above.

Software and code

Policy information about availability of computer code

Data collection	All surface plasmon resonance experiments were performed and analyzed using Biacore T200 Software.<br>All thrombin generation assays were performed and analyzed using Thrombinoscope TM.<br>All thromboelastometry analyses were performed using ROTEM Delta.<br>All scanning electron microscopy images were captured on a FEI Helios NanoLab 650 with user interface xT microscope Control.<br>Intravital microscopy studies were performed and analyzed using the Slidebook program.
Data analysis	All statistical analysis was performed using GraphPad Prism version 9.0 software. Layouts for full sized figures were assembled on Graphpad Prism 9 as well.<br>The ACD/NMR processor spectroscopic software (ACDLABS12.0, ACD/NMR Processor Academic Edition, 1D NMR Processor) was used for NMR data handling.<br>All speciation studies were analyzed using Hyperquad2013 software, and determination of standard electrode potential by a Gran method were performed using GLEE.<br>All fibrin clot thickness studies were analyzed using Image J.<br>Biorender (BioRender.com) for all figures that include mouse doodles.<br>Chemdraw for all chemical structures.

For manuscripts utilizing custom algorithms or software that are central to the research but not yet described in published literature, software must be made available to editors and reviewers. We strongly encourage code deposition in a community repository (e.g. GitHub). See the Nature Portfolio guidelines for submitting code & software for further information.

Data

Policy information about availability of data

All manuscripts must include a data availability statement. This statement should provide the following information, where applicable:

- Accession codes, unique identifiers, or web links for publicly available datasets
- A description of any restrictions on data availability
- For clinical datasets or third party data, please ensure that the statement adheres to our policy

The data that support the findings of this study is available within the main text and its Supplementary Information file. Data is also available from the corresponding author upon request.

Human research participants

Policy information about studies involving human research participants and Sex and Gender in Research.

Reporting on sex and gender	In our study, blood was collected from human volunteers. Adequate representation of males and females were used for the analysis. However, sex specific analysis was not performed in this study. A combined data set is provided in the manuscript.
Population characteristics	Healthy adults (male and female), with no blood-related disorders and normal cell blood counts, 18 years or older, were selected for the study. Care was taken to assure adequate representation of males and females and no individual was excluded on the basis of race or gender. This is not a gender based study.
Recruitment	We used direct recruitment of donors through posted recruitment flyers (no database or contact information stored).The participants were randomly recruited and there was no direct relationship between the study team members and the participants. Care was taken to assure adequate representation of males and females and no individual was excluded on the basis of race or gender. We excluded participants with low hemoglobin level, blood cells, and coagulation disorders, for example, anemia, hemophilia, thalassemia, sickle cell anemia, anemic diseases, as well as, participants taking medication that can interfere with blood cell functions such as Aspirin, and Ibuprofen.
Ethics oversight	The University of British Columbia, Office of Research Ethics, Clinical Research Ethics Board. Procedures involving human subjects in the Centre for Blood Research at the University of British Columbia have been approved by the Institutional Review Board (IRB) within the University of British Columbia (UBC Ethics approval no: H10-01896).

Note that full information on the approval of the study protocol must also be provided in the manuscript.

Field-specific reporting

Please select the one below that is the best fit for your research. If you are not sure, read the appropriate sections before making your selection.

[x] Life sciences [ ] Behavioural & social sciences [ ] Ecological, evolutionary & environmental sciences

For a reference copy of the document with all sections, see nature.com/documents/nr-reporting-summary-flat.pdf

Life sciences study design

All studies must disclose on these points even when the disclosure is negative.

Sample size	For in vitro experiments, sample sizes (N=3 minimum) were chosen based on preliminary experiments and previous laboratory experience from Kizhakkedathu and Morrissey laboratories (Shenoi et al. Sci. Transl. Med. 2014, 6 (260), 260ra150. https://doi.org/10.1126/scitranslmed.3009427 and Travers et al.Blood 2014, 124 (22), 3183–3190. https://doi.org/10.1182/blood-2014-05-577932). For in vivo experiments, sample sizes were chosen based power calculation based on preliminary experiments and prior publications (Shenoi et al. Sci. Transl. Med. 2014, 6 (260), 260ra150. https://doi.org/10.1126/scitranslmed.3009427 and Travers et al.Blood 2014, 124 (22), 3183–3190. https://doi.org/10.1182/blood-2014-05-577932) to arrive at the sample number for different mouse experiments.
Data exclusions	No data was excluded.
Replication	In vitro experiments were performed in biological triplicates and in vivo experiments were repeated upwards of four times (N=/>4) each. Experiments were reproducible.
Randomization	For bleeding studies, randomization was performed by creating an excel sheet listing the samples and controls to be administered per day, using the randomization function (=RAND()) and completing the study on normal C57/BL6 mice with an equal distribution of male and female mice overall in the study. For cremastor arteriole studies, randomization was not applicable as the nature of the study required only male C57/BL6 mice. For saphenous vein hemostasis model both male and female C57/BL6 mice were used. Details are given in the experimental section.
Blinding	Both experimentation and data analysis were blinded to ensure no experimental bias; this was done by creating codes for each sample with

no identifiers.

Reporting for specific materials, systems and methods

We require information from authors about some types of materials, experimental systems and methods used in many studies. Here, indicate whether each material, system or method listed is relevant to your study. If you are not sure if a list item applies to your research, read the appropriate section before selecting a response.

Materials & experimental systems

n/a	Involved in the study
☐	☒ Antibodies
☒	☐ Eukaryotic cell lines
☒	☐ Palaeontology and archaeology
☐	☒ Animals and other organisms
☒	☐ Clinical data
☒	☐ Dual use research of concern

Methods

n/a	Involved in the study
☒	☐ ChIP-seq
☐	☒ Flow cytometry
☒	☐ MRI-based neuroimaging

Antibodies

Antibodies used	DyLight 488-conjugated rat anti-mouse platelet GP1bβ antibody was purchased from EMFRET Analytics.<br><br>Anti-mouse fibrin antibody for in vivo studies was provided by Dr. Rodney M. Camire at Children's Hospital of Philadelphia and Alexa Fluor 647 - conjugated using Alexa Fluor™ Antibody Labeling Kits purchased from Invitrogen based on the manufacturer's instruction.<br><br>Mouse anti-human CD62P-PE antibody BD Biosciences, PE Mouse Anti-Human CD62P, BD Biosciences, cat. no. 550561 (lot 9037907), clone AC1.2, dilution: 1 in 22)<br><br>Anti-CD42a-FITC: FITC Mouse Anti-Human CD42a from BD Biosciences, cat. no. 558818 (lot 9108735), clone ALMA.16, dilution: 1 in 22
Validation	For flow cytometry experiments, antibodies were validated by comparison with any negative control sample and isotypes. The validation of anti-mouse platelet GP1bβ antibody and Anti-mouse fibrin antibody were previously reported (Adili et al Clin. Appl. Thromb. 2021, 27, 107602962110185. https://doi.org/10.1177/10760296211018510 and Adili et al Arterioscler. Thromb. Vasc. Biol. 2017, 37 (10), 1828–1839. https://doi.org/10.1161/ATVBAHA.117.309868).

Animals and other research organisms

Policy information about studies involving animals; ARRIVE guidelines recommended for reporting animal research, and Sex and Gender in Research

Laboratory animals	For bleeding studies, thrombosis models, male and female eight to ten week old C57/BL6 mice were obtained from The Jackson Laboratories (Bar Harbor, ME). Details are given in the experimental section.<br>For intravital microscopy studies, male ten to twelve week old C57/BL6 mice were obtained from The Jackson Laboratories (Bar Harbor, ME). Details are given in the experimental section.<br>For acute and chronic toxicity studies, female six to eight week old Female Balb/cAnNHsd mice Envigo and acclimated 7 days prior to study start.<br>For toxicity studies: Mice are caged in autoclaved Allentown ventilated caging at a capacity of 4 animals/cage during the course of the experiment. Cages are changed bi-weekly. Environmental enrichment that is supplied for cages are Nestlets from Ancare, transparent tinted polycarbonate Mouse Igloos from Bio-Serv on Envigo 7097 ¼” corn cob bedding. All enrichment is added to the cage prior to the cages being autoclaved. Mice are fed Envigo Teklad Global Rodent Diet 2920. The rodent food is kept in the hoppers of the wire lids and is changed bi-weekly. Reverse osmosis water is supplied through Avidity Science automatic watering valves at a flow rate of 25-50ml/min. Environmental control of the lights and monitoring of temperature, humidity and airflow is done by WatchDogEx. Light cycles in the animal holding rooms are set for 12 hours on and 12 hours off. Temperature, humidity and airflow are maintained and controlled by BCCRC (BC Cancer Research Centre) facilities.
Wild animals	This study did not involve wild animals
Reporting on sex	In this study we used both male and female mice. Details are given in the manuscript or in the supplementary information. Sex specific analysis of the data is not reported. A combined data set is given.
Field-collected samples	This study did not involve field-collected samples.

Ethics oversight	Animal bleeding, IVC thrombosis, $FeCl_3$ induced injury model of thrombosis and intravital microscopy experiments were performed in accordance with guidelines and were approved by the University of Michigan Care Committee.<br>Animal toxicity studies were approved by the Institutional Review Board with UBC Ethics approval number A18-0276.

Note that full information on the approval of the study protocol must also be provided in the manuscript.

Flow Cytometry

Plots

Confirm that:

- [x] The axis labels state the marker and fluorochrome used (e.g. CD4-FITC).
- [x] The axis scales are clearly visible. Include numbers along axes only for bottom left plot of group (a 'group' is an analysis of identical markers).
- [x] All plots are contour plots with outliers or pseudocolor plots.
- [x] A numerical value for number of cells or percentage (with statistics) is provided.

Methodology

Sample preparation	Human platelets were used for the analysis. Details are given in the supplementary information.
Instrument	Flow cytometry profiles were acquired using a 3-laser CytoFLEX flow cytometer from Beckman Coulter Life Sciences (10,000 events).
Software	All experimental acquisition and analysis was performed using CytExpert for CytoFLEX Acquisition and Analysis software.
Cell population abundance	A total of 10,000 events were collected for every sample
Gating strategy	An illustration of the gating strategy used for flow cytometry analysis is given in supplementary figure 11 C.

- [x] Tick this box to confirm that a figure exemplifying the gating strategy is provided in the Supplementary Information.
